# A factor converting viable but nonculturable *Vibrio cholerae* to a culturable state in eukaryotic cells is a human catalase

**DOI:** 10.1002/mbo3.264

**Published:** 2015-05-13

**Authors:** Mitsutoshi Senoh, Takashi Hamabata, Yoshifumi Takeda

**Affiliations:** 1 Collaborative Research Center of Okayama University for Infectious Diseases in India, Okayama UniversityKolkata, India; 2 Research Institute, National Center for Global Health and MedicineShinjuku, Tokyo, Japan

**Keywords:** Catalase, factor converting VBNC to culturable, FCVC, VBNC, *Vibrio cholerae*

## Abstract

In our previous work, we demonstrated that viable but nonculturable (VBNC) *Vibrio cholerae* O1 and O139 were converted to culturable by coculture with eukaryotic cells. Furthermore, we isolated a factor converting VBNC *V. cholerae* to culturable (FCVC) from a eukaryotic cell line, HT-29. In this study, we purified FCVC by successive column chromatographies comprising UNO Q-6 anion exchange, Bio-Scale CHT2-1 hydroxyapatite, and Superdex 200 10/300 GL. Homogeneity of the purified FCVC was demonstrated by SDS-PAGE. Nano-LC MS/MS analysis showed that the purified FCVC was a human catalase. An experiment of RNAi knockdown of catalase mRNA from HT-29 cells and treatment of the purified FCVC with a catalase inhibitor, 3-amino-1,2,4-triazole confirmed that the FCVC was a catalase. A possible role of the catalase in converting a VBNC *V. cholerae* to a culturable state in the human intestine is discussed.

## Introduction

The viable but nonculturable (VBNC) state of bacteria is defined as bacteria that are alive, but not growing or dividing on/in the routinely used bacteriological media. Many bacteria, comprising more than 60 species including a large number of human pathogens, are known to enter the VBNC state in response to natural stresses such as starvation and fluctuation in temperature or osmotic concentration (Oliver 2005, 2010; Pinto et al. 2013; Li et al. 2014). Following the first report of VBNC *Vibrio cholerae* by Xu et al. (1982), many investigators have reported studies on VBNC *V. cholerae* (Colwell et al. 1985; Kondo et al. 1994; Binsztein et al. 2004; Islam et al. 2004; Asakura et al. 2006; Faruque et al. 2006; González-Escalona et al. 2006; Alam et al. 2007; Aulet et al. 2007; Halpern et al. 2007; Mishra et al. 2011).

Colwell et al. (1985) reported the conversion/resuscitation of VBNC *V. cholerae* to a culturable state by inoculation into a rabbit ileal loop and by administration to human volunteers (Colwell et al. 1996). Wai et al. (1996) reported that a heat shock of VBNC *V. cholerae* could convert the VBNC state to a culturable state. Recently Bari et al. (2013) reported that culturable *V. cholerae* were recovered from environmental water samples by addition of the quorum-sensing molecules CAI-1 and AI-2 suggesting that VBNC *V. cholerae* in the environmental water samples were converted/resuscitated to culturable by these molecules. AI-2 was also able to induce conversion/resuscitation of other VBNC vibrios, such as VBNC *Vibrio vulnificus*, to culturable state (Ayrapetyan et al. 2014).

Previously, we demonstrated the conversion/resuscitation of the VBNC state of *V. cholerae* O1 and O139 and some other enteric bacteria to a culturable state by coculture with various eukaryotic cells, including the human colonic epithelial cell line HT-29 (Senoh et al. 2010, 2012). Subsequently, we extracted a factor converting VBNC *V. cholerae* to culturable (FCVC) from HT-29 cells and demonstrated that FCVC had a proteinaceous nature (Senoh et al. 2014). In this study, we performed a characterization of FCVC and identified it as a catalase.

## Materials and Methods

### Bacterial strains and culture media

*Vibrio cholerae* O1 (N16961) and O139 (VC-280) were from stock cultures in the laboratory repository at the National Institute of Cholera and Enteric Diseases. The culture media used were alkaline peptone water (APW) pH 8.8 (Eiken, Tokyo, Japan), nutrient agar (Difco, Franklin Lakes, NJ) supplemented with 1% NaCl (NA) and thiosulfate citrate bile salts sucrose agar (TCBS) (Eiken).

### Preparation of VBNC *V. cholerae*

VBNC *V. cholerae* O1 (N16961) and O139 (VC-280) were prepared as described previously (Senoh et al. 2010, 2014). Each *V. cholerae* strain was inoculated into APW and incubated at 37°C for 16 h. Subsequently, the cells were collected by centrifugation at 5000*g* for 10 min at 25°C (Heraeus Biofuges Strators; Kendro, Langenselbold, Germany), washed twice with a VBNC microcosm buffer comprising 1% sterile solution of artificial seawater (1% Instant Ocean; Aquarium Systems, Mentor, OH), and suspended in 200 mL of VBNC microcosm buffer in a 1-L flask to a final concentration of approximately 1 × 10^8^ cells mL^−1^. The cells in VBNC microcosm buffer were incubated at 4°C, in the dark, without shaking. After incubation for 11 weeks, no culturable cells were observed after incubation of VBNC microcosm buffer in APW at 37°C for 16 h.

### Culture of HT-29 cell

HT-29 eukaryotic cells (human colon adenocarcinoma grade II cells) were cultured in Dulbecco’s modified Eagle’s medium (catalogue number: 12800-017; Gibco Life Science, Paisley, Scotland, UK) supplemented with 1.5 g L^−1^ NaHCO_3_ (Sigma, St. Louis, MO), 3.56 g L^−1^ 4-(2-hydroxyethyl)-1-piperazine-ethanesulfonic acid (Sigma), 10% fetal bovine serum (FBS; catalogue number 10082-147; Gibco Life Science), 100 *μ*g mL^−1^ streptomycin and 100 U mL^−1^ penicillin. All cell cultures were carried out in a CO_2_ incubator at 37°C under 5% CO_2_.

### Preparation of cell extracts

Confluent HT-29 cells in 10-cm in diameter petri dishes were washed with phosphate-buffered saline (PBS), scraped off, and centrifuged at 3000*g* for 5 min at 25°C. The collected cells were resuspended in 0.5 mL of PBS, mixed with 0.1-mm in diameter glass beads and disrupted by shaking for 90 sec using a ShakeMaster (BioMedical Science, Tokyo, Japan). After centrifugation of the mixture at 20,000*g* for 5 min at 4°C, the supernatant was passed through a 0.22-*μ*m membrane filter (EMD Millipore Corporation, Billerica, MA). The obtained cell extract was designated a solution of the factor converting VBNC to culturable (FCVC).

### Examination of the activity of FCVC to convert VBNC *V. cholerae* to a culturable state

A 0.1-mL aliquot of a VBNC *V. cholerae* and 0.05 mL of a twofold serially-diluted FCVC preparation were added to 0.3 mL of 1.5-fold condensed APW and incubated at 37°C for 16 h without shaking. When the APW became turbid, a 0.1-mL aliquot was inoculated onto TCBS plates and incubated at 37°C for 16 h. The yellow colonies that appeared were inoculated onto NA plates and incubated at 37°C for 16 h. The serotypes of the colonies that appeared on the NA plates were confirmed with appropriate typing sera (Denka, Tokyo, Japan). The activity of FCVC was expressed by the reciprocal of the highest dilution of the FCVC that converted VBNC *V. cholerae* to a culturable state. It was confirmed in each experiment that VBNC *V. cholerae* did not grow by incubation at 37°C for 16 h in APW without FCVC, as the negative control.

### Purification of FCVC

Aliquots (250 mL) of the cell extracts containing the FCVC solution, as prepared above, were centrifuged at 100,000*g* for 1 h at 4°C, and 0.176 g mL^−1^ of ammonium sulfate was added to the supernatants. The mixtures were centrifuged at 7000*g* for 20 min, and the resulting precipitates were discarded. The supernatants were mixed with 0.125 g mL^−1^ of ammonium sulfate and then centrifuged at 7000*g* for 20 min. The resulting precipitates were dissolved in 5 mL of PBS. The FCVC solution thus prepared was placed in a dialysis bag (SnakeSkin®; MWCO: 10,000; Takara Bio Inc., Shiga, Japan) and dialyzed against 2 L of 20 mmol L^−1^ Tris-HCl (pH 8.5) at 4°C for 16 h.

The crude FCVC solution was first applied to a 12 × 53 mm column of UNO Q-6 anion exchange (Bio-Rad, Hercules, CA) previously equilibrated with 20 mmol L^−1^ Tris-HCl buffer (pH 8.5). The protein bound to the matrix was eluted with a linear NaCl gradient of 0–0.5 mol/L NaCl at a flow rate of 1 mL min^−1^. The fractions containing the FCVC activity were collected, concentrated by centrifugation at 8000*g* for 10 min at 4°C with VIVASPIN 20 (MWCO: 50,000; Sartorius AG, Goettingen, Germany), and suspended in 10 mmol L^−1^ phosphate buffer (pH 6.8). The sample was applied to a 7 × 52 mm column of Bio-Scale CHT2-1 hydroxyapatite previously equilibrated with 10 mmol L^−1^ phosphate buffer (pH 6.8). The protein bound to the matrix was eluted with a linear gradient of 10–500 mmol L^−1^ phosphate buffer (pH 6.8) at a flow rate of 1 mL min^−1^. The fractions containing the FCVC activity were collected, concentrated by centrifugation at 8000*g* for 10 min at 4°C with VIVASPIN 20 (MWCO: 50,000), and suspended in 20 mmol L^−1^ Tris-HCl buffer (pH 8.5). The sample was then applied to a 10 × 300 mm column of Superdex 200 10/300 GL (GE Healthcare, Uppsala, Sweden) previously equilibrated with 20 mmol L^−1^ Tris-HCl buffer (pH 8.5). The protein bound to the matrix was eluted with 20 mmol L^−1^ Tris-HCl buffer (pH 8.5) containing 200 mmol L^−1^ NaCl at a flow rate of 0.5 mL min^−1^. The fractions containing the FCVC activity were collected, concentrated by centrifugation at 8000*g* for 10 min at 4°C with VIVASPIN 20 (MWCO: 50,000), and suspended in 20 mmol L^−1^ Tris-HCl buffer (pH 8.5). The Superdex 200 10/300 GL column chromatography was repeated under the same condition.

### Protein concentration

The protein concentration was determined using a Bradford protein estimation kit (Bio-Rad).

### SDS-PAGE

Sodium dodecyl sulfate polyacrylamide gel electrophoresis (SDS-PAGE) with 0.1% SDS was carried out as described by Laemmli (1970) using a 7.5% acrylamide gel. The samples were heated at 100°C for 5 min in the presence of 1% SDS and then electrophoresed at a constant current of 30 mA for 1 h at room temperature. The resulting gels were stained with EzStain Silver (Atto, Tokyo, Japan). Molecular weight markers were purchased from Bio-Rad.

### Nano-LC MS/MS analysis

The purified band of FCVC was excised from the polyacrylamide gels and subjected to nano-LC MS/MS analysis (Japan Bio Services Co., Ltd., Saitama, Japan). The MS data were analyzed by a Mascot search against a peptide sequence database (NCBInr 20110417).

### Fractionation of HT-29 cells

Confluent HT-29 cells in 10-cm-diameter petri dishes were fractionated into cytosolic, membranes and organelles, nuclear, and cytoskeleton fractions using a Proteo Extract Subcellular Proteome Extraction Kit (Merck, Darmstadt, Germany), in accordance with the manufacturer’s instructions. Each fraction was passed through a 0.22-*μ*m filter (EMD Millipore Corporation).

### RNAi knockdown of catalase mRNA

A Stealth short-interfering RNA (siRNA; Invitrogen, Carlsbad, CA) against catalase (CATHSS101395) was tested in HT-29 cells. Approximately 1 × 10^6^ HT-29 cells in 10-cm in diameter petri dishes were transfected with 50 nmol L^−1^ of siRNA and Lipofectamine RNAiMAX transfection reagent (Invitrogen). After incubation at 37°C for 72 h under 5% CO_2_, the cells were washed with PBS, scrapped off, and centrifuged at 3000*g* for 5 min at 25°C. The collected cells were resuspended in 0.5 mL of PBS, mixed with 0.1-mm-diameter glass beads and disrupted by shaking for 90 sec using a ShakeMaster (BioMedical Science). After centrifugation of the mixture at 20,000*g* for 5 min at 4°C, the supernatant was passed through a 0.22-*μ*m membrane filter (EMD Millipore Corporation).

### Treatment with 3-amino-1,2,4-triazole

The purified FCVC was mixed with 3-amino-1,2,4-triazole (final concentration 50 nmol L^−1^; Sigma), and incubated at 4°C for 2 h. The mixed sample was subjected to ultrafiltration using an Amicon Ultra (MWCO: 10,000; EMD Millipore Corporation) to remove the 3-amino-1,2,4-triazole. After two washes with PBS, the sample was resuspended in PBS and passed through a 0.22-*μ*m membrane filter (EMD Millipore Corporation).

### Measurement of catalase activity

Catalase activity was measured using a Catalase Assay Kit (Sigma) in accordance with the manufacturer’s instructions.

## Results

Cell extracts of a eukaryotic cell, HT-29, were prepared and FCVC in the cell extracts was purified by ammonium sulfate fractionation and successive column chromatographies comprising anion exchange, hydroxyapatite, and gel filtration (Fig. S1). The FCVC activity to convert VBNC *V. cholerae* to a culturable state was followed during each step of the purification. As shown in Table1, from a total of 64,000 U of FCVC activity in a cell extract, 80 U of the purified FCVC was recovered through several purification steps, providing a 0.125% yield of the activity. The specific activity of the FCVC activity increased from 90 U/mg in the cell extract to 80,000 U/mg in the final preparation. The total protein content of the purified FCVC was 0.001 mg.

**Table 1:** Purification of FCVC

Step	Total protein (mg)	Total activity^1^ (U)	Specific activity (U/mg)	Yield (%)
Cell extract	710	64,000	90	100
Ultracentrifugation	534	62,080	116	97
Salting out (30–50%)	207	36,800	178	58
Anion exchange chromatography (UNO Q-6)	7.6	11,520	1518	18
Hydroxyapatite chromatography (CHT2-1)	0.54	3840	7111	6
First gel filtration chromatography (SD200 10/300 GL)	0.02	1280	64,000	2
Second gel filtration chromatography (SD200 10/300 GL)	0.001	80	80,000	0.125

1 The activity of FCVC to convert VBNC *Vibrio cholerae* to a culturable state was measured as described in the text.

The purified FCVC and samples at each purification step were subjected to SDS-PAGE to examine the homogeneity of the purified material. The purified FCVC resulted in a single band in the SDS-PAGE gel (Fig. S2), indicating FCVC was purified to homogeneity.

Nano-LC MS/MS analysis of the material excised from the single band on the SDS-PAGE gel detected 65 peptides that covered 61% of human catalase (Fig. S3).

**Figure 1:**
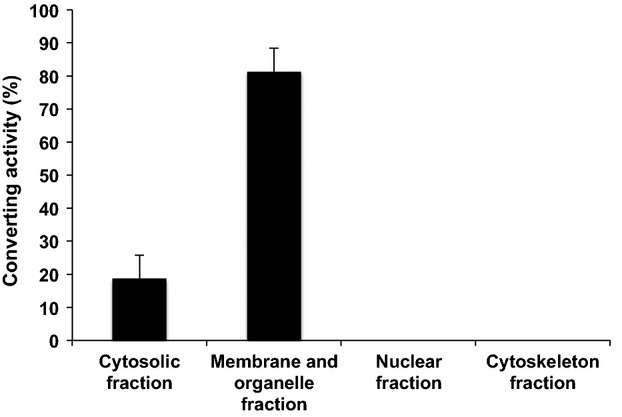
Converting activity of several fractions of HT-29 cells. HT-29 cell extracts were fractionated, and the converting activity of each fraction was measured as described in the text. The converting activity was expressed as a percentage of the total activity in the cell extract. Bars represent means ± SD of three determinations.

To examine whether FCVC has catalase activity, we fractionated the cellular proteins of HT-29 cells and measured their VBNC-converting activity. As shown in Figure1, the converting activity was localized mostly in the membrane and organelle fraction and slightly in the cytosolic fraction, with no activity in the nuclear and cytoskeleton fractions. These results are consistent with the fact that catalase is located in peroxisomes within cells (De Duve and Baudhuin 1966).

**Figure 2:**
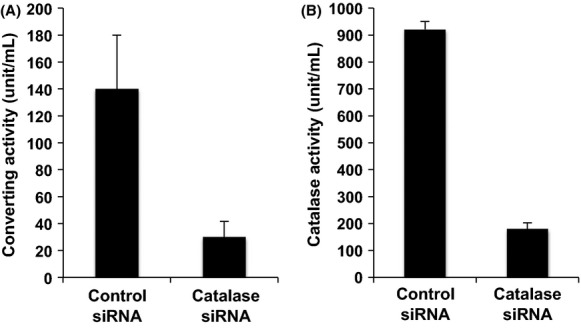
Activity of cell extracts from catalase siRNA-transfected HT-29 cells. Cell extracts of catalase siRNA-transfected HT-29 cells were prepared, and measured for their converting activity (A) and catalase activity (B) were measured as described in the text. The culturability of VBNC *Vibrio cholerae* by incubation at 37°C for 16 h in APW without FCVC was not detected. Bars represent means ± SD of four determinations.

To obtain further evidence, we employed an siRNA to knockdown the catalase expression in HT-29 cells and examine the effect on the VBNC-converting activity of the purified FCVC. As shown in Figure2A, siRNA-induced reduction of catalase expression resulted in a significant decrease in the FCVC activity. The rate of decrease in the FCVC activity shown in Figure2A was almost the same as the decrease in the H_2_O_2_-degrading activity (catalase activity) shown in Figure2B.

**Figure 3:**
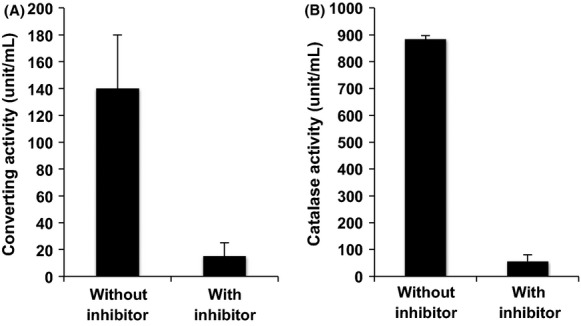
Inhibition of the converting activity of the purified FCVC with 3-amino-1,2,4-trizole. The converting activity (A) and catalase activity (B) of the purified FCVC with and without the inhibitor 3-amino-1,2,4-trizole were measured as described in the text. The culturability of VBNC *Vibrio cholerae* by incubation at 37°C for 16 h in APW without FCVC was not detected. Bars represent means ± SD of four determinations.

In the experiment shown in Figure3, we examined the effect of a catalase inhibitor, 3-amino-1,2,4-triazole, on the VBNC-converting activity of the purified FCVC. The presence of 3-amino-1,2,4-triazole significantly decreased the VBNC-converting activity (Fig.3A) as well as the H_2_O_2_-degrading activity of FCVC (Fig.3B) of the purified FCVC.

## Discussion

In cholera endemic areas, such as Kolkata in India, VBNC *V. cholerae* O1 and O139 were found in environmental water samples in both epidemic and nonepidemic seasons (Faruque et al. 2006; Alam et al. 2007; Bari et al. 2013; Senoh et al. 2014). It has been assumed that such VBNC *V. cholerae* O1 and O139 in environmental water act as a source of infection in these areas. To further examine the possible significance of the VBNC *V. cholerae* as a source of the infection, it is necessary to clarify the mechanism underlying the conversion/resuscitation of the VBNC state of bacteria to a culturable state. However, little is known about the mechanism for the conversion/resuscitation of VBNC *V. cholerae* to a culturable state.

Several conditions or factors for conversion/resuscitation have been reported, such as temperature upshift for VBNC *V. vulnificus* and *Salmonella enteritica* serovar Typhumurium (Whitesides and Oliver 1997; Gupte et al. 2003), heat-stable autoinducer of growth for VBNC *Escherichia coli* (Reissbrodt et al. 2002), resuscitation-promoting factor for VBNC *S. enteritica* serovar Oranienburg (Panutdaporn et al. 2006), presence of *Acanthamoeba castellanii* for VBNC *Legionella pneumophila* (Steinert et al. 1997), and incubation in phosphate buffer for VBNC *E. coli* (Dukan et al. 1997). We reported that coculture with eukaryotic cells led to the conversion/resuscitation of VBNC *V. cholerae* (Senoh et al. 2010) and several enteric bacteria (Senoh et al. 2012). Furthermore, we identified FCVC, a factor converting VBNC *V. cholerae* to a culturable state in cell extracts of eukaryotic cells (Senoh et al. 2014).

In this study, FCVC extracted from HT-29 cells, which has a proteinaceous nature, was purified to homogeneity by successive column chromatographies (as evaluated by SDS-PAGE), and identified as a catalase by nano-LC MS/MS analysis. Several experiments including RNAi knockdown in HT-29 cells and treatment of FCVC with the catalase inhibitor, 3-amino-1,2,4-triazole confirmed that FCVC is a human catalase.

There are several contradictory reports on the effects of catalase for the conversion/resuscitation of VBNC bacteria to a culturable state. Wai et al. (2000) and Mizunoe et al. (2000) reported that catalase converted/resuscitated VBNC *Aeromonas hydrophila* and *Vibiro parahaemolyticus*, respectively. Imazaki and Nakaho (2009) reported that *Ralstonia solanacearum*, a plant pathogen, exhibits two types of VBNC state: a sodium pyruvate (SP)-recoverable VBNC state that can be recovered by H_2_O_2_-degrading compounds such as SP and catalase, and an SP-unrecoverable VBNC state. Conversely, Lleò et al. (2001) showed no effect of catalase on the resuscitation of VBNC enterococcal strains, and Gupte et al. (2003) reported that catalase was not effective for the resuscitation of VBNC *Salmonella enterica* serovar Typhimurium.

In vivo conversion/resuscitation of VBNC *V. cholerae* was first reported by Colwell and colleagues in a rabbit ileal loop test (Colwell et al. 1985) and human volunteer studies (Colwell et al. 1996). Du et al. (2007) demonstrated the conversion/resuscitation of VBNC *Edwardsiella tarda* in chicken embryo. However, little is known about the possible mechanism for the conversion/resuscitation under these in vivo conditions. Our findings that VBNC *V. cholerae* and other enteric bacteria were converted/resuscitated to a culturable state by coculture with various eukaryotic cells (Senoh et al. 2010, 2012) may mimic in vivo conditions, and we therefore tried to identify a factor for the conversion/resuscitation from eukaryotic cells in this study.

Previously we reported the successful isolation of VBNC *V. cholerae* O1 El Tor variant strains harboring a gene for the cholera toxin from environmental water samples collected in urban slum areas of Kolkata, India (Senoh et al. 2014). Together with the results of this study, we suggest that VBNC *V. cholerae* in environmental water in cholera-endemic areas will be converted/resuscitated to a culturable state in the human intestine by the action of catalase and subsequently cause the disease, cholera.

## Supporting Information

**Figure S1.** Column chromatograms of the crude FCVC. Successive column chromatographies of the crude FCVC on UNO Q-6 anion exchange, Bio-Scale CHT2-1 and Superdex 200 10/300 GL were carried out as described in the text. Typical column chromatograms of (A) UNO Q-6 anion exchange, (B) Bio-Scale CHT2-1, (C) first Superdex 200 10/300 GL and (D) second Superdex 200 10/300 GL are shown. The converting activity of each fraction was measured as described in the text.

**Figure S2.** SDS-PAGE of FCVC. SDS-PAGE was carried out as described in the text. (M) Molecular weight markers. Lane 1, 28.4 *μ*g of cell extracts; lane 2, 22.0 *μ*g of cell extracts after ultracentrifugation; lane 3, 20.7 *μ*g of crude FCVC; lane 4, 7.6 *μ*g of FCVC after UNO Q-6 anion exchange column chromatography; lane 5, 1.8 *μ*g of FCVC after Bio-Scale CHT2-1 column chromatography; lane 6, 0.2 *μ*g of FCVC after first Superdex 200 10/300 GL column chromatography; lane 7, 0.01 *μ*g of FCVC after second Superdex 200 10/300 GL column chromatography.

**Figure S3.** Peptide sequences of the purified FCVC. After nano-LC MS/MS analysis of the purified FCVC, the MS data were analyzed as described in the text, and 65 peptides were identified in the amino acid sequence (shown in single letters) of human catalase. The matched peptide sequences are indicated in thick bold typeface, and covered 61% of the entire sequence. The numbers of the amino acid residue are given on the left.
