# Supplementary figures and images for: A factor converting viable but nonculturable Vibrio cholerae to a culturable state in eukaryotic cells is a human catalase
Source: Microbiologyopen. 2015 May 13;4(4):589–96. doi: 10.1002/mbo3.264 (PMC4554454; doi:10.1002/mbo3.264)

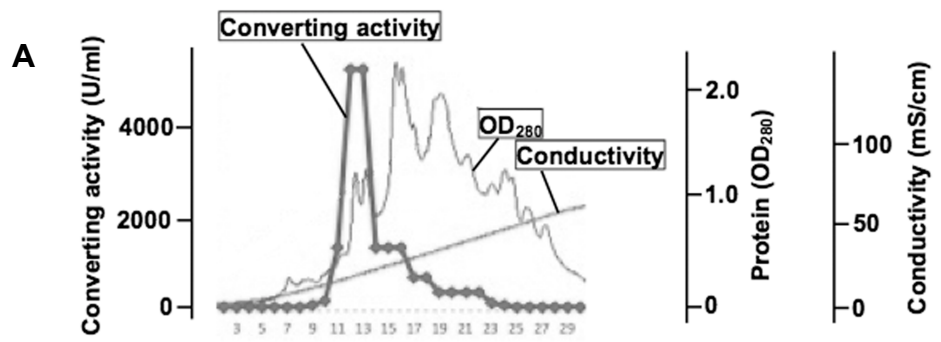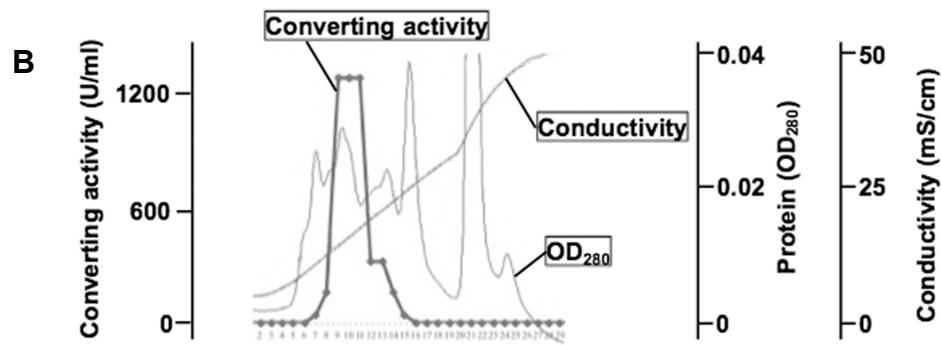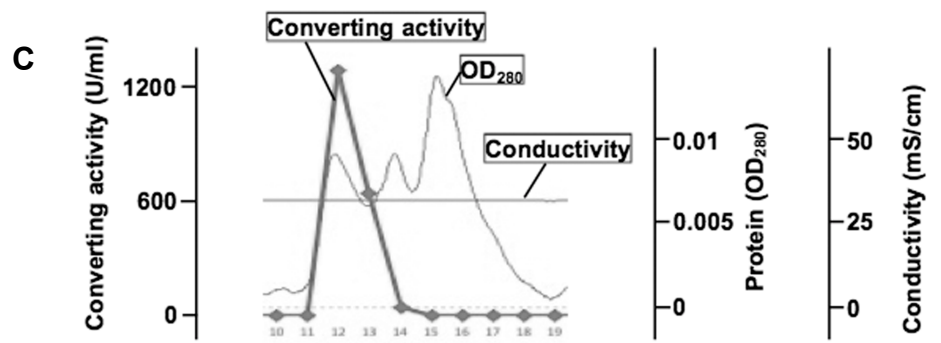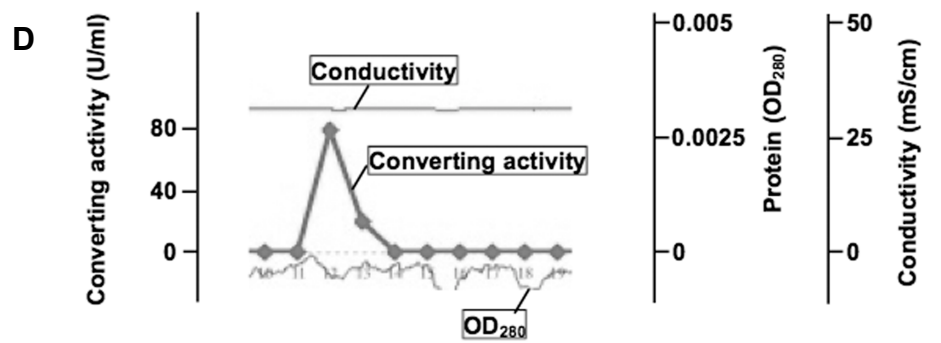

Supplement: Supplementary file 1 [file mbo30004-0589-sd1.pdf]

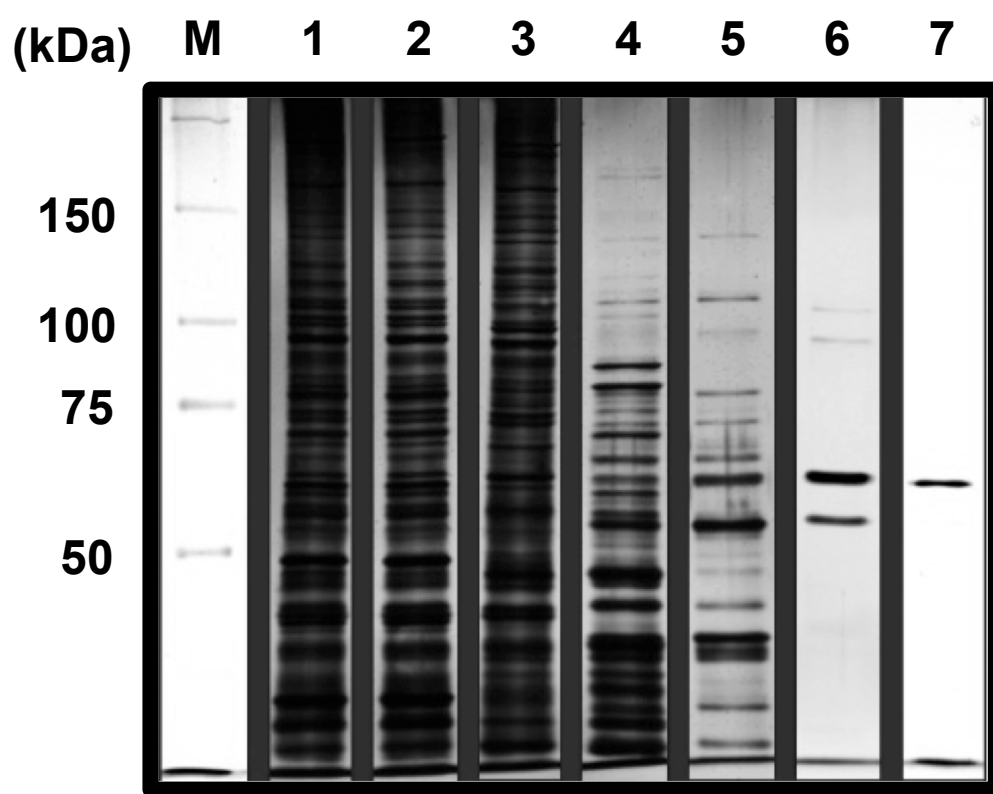

Supplement: Supplementary file 2 [file mbo30004-0589-sd2.pdf]
