# Supplementary material for: A factor converting viable but nonculturable Vibrio cholerae to a culturable state in eukaryotic cells is a human catalase
Source: Microbiologyopen. 2015 May 13;4(4):589–96. doi: 10.1002/mbo3.264 (PMC4554454; doi:10.1002/mbo3.264)
Supplement: Supplementary file 3 [file mbo30004-0589-sd3.pdf]

1 **MADSR**DPASD QMQHWKEQR**A** **AQKADVLTTG** **AGNPVGDKLN** **VITVGPRGPL**
51 **LVQDVVFTDE** **MAHFDRER**IP ERVVHAK**GAG** **AFGYFEVTHD** **ITK**YSKAKVF
101 EHIGKKTPIA VR**FSTVAGES** **GSADTVRDPR** GFAVK**FYTED** **GNWDLVGNNT**
151 **PIFFIRDPIL** **FPSFIHSQKR** NPQTHLKDPD MVWDFWSLRP ESLHQVSFLF
201 SDRGIPDGHR **HMNGYGSHTF** **KLVNANGEAV** **YCK**FHYKTDQ GIK**NLSVEDA**
251 **AR**LSQEDPDY GIR**DLFNAIA** **TGK**YPSWTFY IQVMTFNQAE TFPFNPFDLT
301 K**VWPHKDYPL** **IPVGK**LVLNR **NPVNYFAEVE** **QIAFDPSNMP** **PGIEASPDK**M
351 LQGR**LFAYPD** **THRHRLGPNY** **LHIPVNCPYR** ARVANYQRDG PMCMQDNQGG
401 APNYYPNSFG APEQQPSALE HSIQYSGEVR **RFNTANDDNV** **TQVRAFYVNV**
451 **LNEEQRKRLC** **ENIAGHLKDA** **QIFIQK**KAVK **NFTEVHPDYG** **SHIQALLDKY**
501 **NAEKPKNAIH** **TFVQSGSHLA** **AR**EKANL
